# Bullous Congenital Ichthyosiform Erythroderma with Tinea Capitis in Half-Siblings: Rare Phenomenon in Ichthyosis with Co-Existing *Trichophyton rubrum* Infection and Blocker Displacement Amplification for Mosaic Mutation Detection

**DOI:** 10.3390/biomedicines13082015

**Published:** 2025-08-19

**Authors:** Jipeng Liu, Yujuan Fu, Qihao Zhang, Qi Chen, Yuxiang Yang, Yi Xue, Yunqing Ren

**Affiliations:** Department of Dermatology, Children’s Hospital, Zhejiang University School of Medicine, National Clinical Research Center for Child Health, Hangzhou 310052, China; liujipeng@zju.edu.cn (J.L.); 2519151@zju.edu.cn (Y.F.); zhangqh2024zju@163.com (Q.Z.); 22418784@zju.edu.cn (Q.C.); yyx1978984350@163.com (Y.Y.); yxue@zju.edu.cn (Y.X.)

**Keywords:** bullous congenital ichthyosiform erythroderma, tinea capitis, comorbidity, blocker displacement amplification

## Abstract

**Background/Objectives**: Bullous congenital ichthyosiform erythroderma (BCIE) is an inherited keratinization disorder caused by pathogenic variants in specific genes. Here, we report a pair of half-siblings with BCIE and tinea capitis due to *Trichophyton rubrum* (*T. rubrum*) and then review the species of ichthyosis previously reported with *T. rubrum* infection. **Methods**: We performed dermatological examination, fungal culture, and genetic analysis using whole-exome sequencing (WES) and blocker displacement amplification (BDA)-based Sanger sequencing. Both patients received oral terbinafine once daily and topical bifonazole gel for tinea capitis. **Results**: The pair of half-siblings had exhibited generalized scaling and hyperkeratosis since birth. Both siblings subsequently developed scalp pustules and hair loss for several months. Genetic analysis identified a pathogenic variant in the keratin 10 (*KRT10*) gene, confirming BCIE diagnosis. Additionally, fungal culture revealed *T. rubrum* infection. The patients responded positively to oral terbinafine antifungal treatment. **Conclusions**: This case highlights the potential susceptibility of patients with BCIE to fungal infections, warranting clinical vigilance. Furthermore, it demonstrates the utility of the BDA-based mutation detection method for diagnosing BCIE, suggesting its promise for advancing personalized diagnosis and management in hereditary skin diseases.

## 1. Introduction

Bullous congenital ichthyosiform erythroderma (BCIE), also known as epidermolytic hyperkeratosis (EHK) [1] and epidermal differentiation disorder (EDD) [2], is a rare autosomal dominant disorder caused by mutations in the keratin 1 (*KRT1*) or keratin 10 (*KRT10*) genes, encoding structural proteins essential for epidermal integrity [3]. Affected newborns present with marked hyperkeratosis and moist and fragile erythematous skin, which leads to extensive blister formation, focal aggregates of tonofilaments, and cytolysis within terminally differentiated epidermal cells [4]. The prevalence is approximately 1 in 300,000 infants [1]. With age, blistering and erythema typically diminish, giving way to verrucous hyperkeratosis. In adulthood, it is characterized by hyperkeratotic lesions, often accompanied by erosions and vesicles. These primary dermatological features, particularly xerosis, pruritus, and rhagades, extend beyond cosmetic concerns to encompass significant psychosocial sequelae, including stress disorders and impaired self-perception [5].

Previous studies indicate a high frequency (50–75%) of de novo *KRT1* or *KRT10* mutations [6,7]. A proportion of these de novo mutations represent undetected parental mosaicism, as the low mutant allele fraction often evades detection by conventional Sanger sequencing [8]. Furthermore, BCIE cases can also arise from somatic mutations in only *KRT1* or *KRT10* [9]. Recent studies demonstrate that the highly sensitive blocker displacement amplification (BDA) method exploits differences in probe hybridization affinity between variant and wild-type templates, achieving distinct differential amplification yields [10]. This enhances the detection sensitivity for somatic mosaicism in genetic diagnostics [11].

Dermatophytes, such as the ubiquitous *Trichophyton rubrum* (*T. rubrum*) [12], are fungi that utilize keratinized tissues (skin, hair, nails) for nutrients [13]. While both dermatophytosis and ichthyosis are common, co-infection with *T. rubrum* has been documented primarily in ichthyosis vulgaris [14,15], with relatively few reports in hereditary ichthyoses like BCIE. However, an association between the two conditions is biologically plausible, potentially attributable to the immunological and skin barrier defects inherent to ichthyoses.

Here, we report tinea capitis in two siblings with BCIE. Our study aims to highlight the importance of considering mosaic mutations in BCIE patients and raise awareness of their potential susceptibility to dermatophytosis, necessitating close clinical monitoring.

## 2. Materials and Methods

### 2.1. Ethics Statement

Written informed consents were obtained from all parents. The study was approved by the Ethics Committee of the Children’s Hospital, Zhejiang University School of Medicine (No.2022-IRB-046), and conducted in accordance with the Declaration of Helsinki.

### 2.2. Sample Collection and DNA Extraction

Peripheral blood samples were collected in EDTA tubes from both patients and their parents. Genomic DNA was extracted using the QIAamp DNA Blood Mini Kit (QIAGEN, Hilden, Germany). Somatic cells from the mouth and hair of the parents and germ cells of the father were collected. In brief, after rinsing the mouth twice with purified water to remove debris, buccal mucosa cells were collected by vigorously swabbing the buccal mucosa 15 times bilaterally using sterile cotton swabs. DNA was extracted using the TIANamp Genomic DNA kit (TIANGEN, Beijing, China). Approximately 20 hairs with intact follicular bulbs were plucked per individual. DNA was extracted using the TIANamp Micro DNA Kit (TIANGEN, Beijing, China).

### 2.3. Whole Exome Sequencing

Whole exome sequencing (WES) was performed on the proband. DNA library preparation and exome capture were conducted using the NEB Next DNA Library Prep Master Mix Set for Illumina (NEB, Ipswich, MA, USA) and Nano WES V1 according to the manufacturer’s protocol. The exome library was sequenced on the Illumina Novaseq 6000 platform v 1.7 (Illumina, San Diego, CA, USA) and aligned to the hg38/GRCh38 human reference sequence. The functions of the detected variants were annotated in ANNOVAR software (v 2020Jun08).

### 2.4. Conventional PCR

The primers used in study were designed using Primer3Plus (http://www.primer3plus.com, accessed on 16 April 2025). The primers used in conventional PCR are forward: 5′- GCAGTTTCGGAGGTGGCA -3′; reverse: 5′- GTAGTATTTGCTGTAGTCACGAGGC -3′. The conventional PCR was performed by PrimeSTAR^®^ Max DNA Polymerase (Takara, Dalian, China), and the amplification conditions for conventional PCR were 95 °C for 2 min, 98 °C for 10 s, 60 °C for 30 s, and 72 °C for 10 s for 35 cycles, according to the manufacturer’s protocol. The products were then confirmed by 0.5% agarose gel electrophoresis and used for Sanger sequencing.

### 2.5. Blocker Displacement Amplification-Based PCR

Variants identified in the *KRT10* gene as putatively disease-causative were verified with blocker displacement amplification (BDA), preventing the false positivity due to the interference of pseudogenes or mismatches in long segment amplification. The primers and blockers used in BDA-based PCR are forward: 5′- CCCGAACTTTGTCCAAGTAGGAA-3′; reverse: 5′- CAGGAAACAGCTATGACCGACTTCGGAGGTGGCAGCTTTG -3′; blocker: 5′- GTAGGAAGCCAGGCGGTCATTCAGATGAGAACATTAGTTCTCGTTAGCAATAAC -3′. The DNA samples for patients and their parents were tested with blocker (standard BDA) and without blocker (forward and reverse primers only). The qPCR assays were performed using PowerUp SYBR Green Master Mix (Thermo Fisher Scientific, Waltham, MA, USA), with 400 nmol/L of each primer, 4 mmol/L of blocker, and 10 ng of DNA per well. Reactions were performed in the final volume of 30 µL per well using the CFX96 Touch Real-Time PCR Detection System (Bio-Rad, Hercules, CA, USA). The amplification conditions for BDA-based PCR were 95 °C for 3 min, 95 °C for 30 s, and 60 °C for 30 s for 40 cycles. The short extension time prevented amplification of longer, potentially nonspecific amplicons. Each qPCR was repeated at least twice. Cq values generated by qPCR were used to aid in the determination of the presence of low levels of mosaic mutations. Detailed interpretations can be made according to the previously developed protocol [16].

### 2.6. Sanger Sequencing

Sanger sequencing was used to confirm the candidate mutations identified during WES. The amplified PCR products were purified using a QIAQuick PCR purification kit (QIAGEN, Hilden, Germany) and directly sequenced on an ABI PRISM3730XL automated sequencer (Applied Biosystems, Thermo Fisher Scientific, Waltham, MA, USA). The Chromas v2.6.6 was used to display the signal for each base site. Each base is represented using a different color, and the height of the peak indicates the signal intensity of the base.

### 2.7. Statistical Analysis

To verify whether the variants were previously recorded, we searched the variants in the dbSNP (http://www.ncbi.nlm.nih.gov/projects/SNP/, accessed on 16 April 2025), 1000 genomes (1000G, https://www.internationalgenome.org/, accessed on 16 April 2025), Human Gene Mutation Database (HGMD, http://www.hgmd.cf.ac.uk/, accessed on 16 April 2025), Human Exon Database (ExAC, http://exac.broadinstitute.org, accessed on 16 April 2025), gene database, and the Population Genome Mutation Frequency Database (gnomAD, http://gnomad-sg.org/, accessed on 16 April 2025). Variants were identified as novel if they had never been reported in the literature and were absent from the database.

## 3. Results

### 3.1. Clinical Manifestations

**Figure 1:**
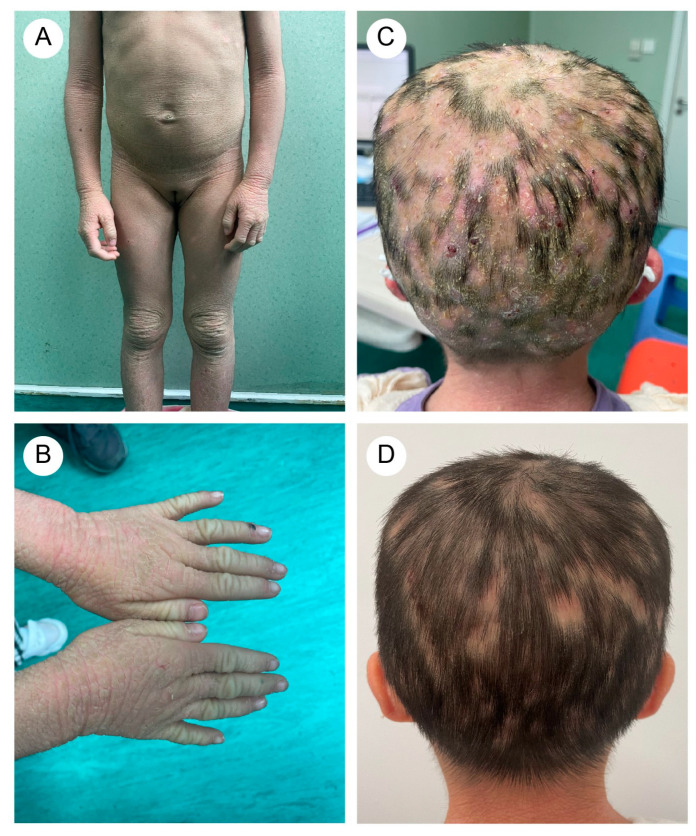
(**A**) The proband (older sister) exhibited rough, grayish skin throughout the body, with pronounced keratosis on the limbs and trunk, particularly around the joints. (**B**) Excessive keratotic erythema on the hands. (**C**) Widespread distribution of pustular folliculitis on the scalp, accompanied by multiple patches of hair loss (occipital region). (**D**) After treatment, the erythematous pustules on the scalp subsided, leaving areas of moth-eaten alopecia.

A five-year-and-eight-month-old Chinese girl presented with generalized pustules on the scalp accompanied by multifocal alopecia. Her medical history revealed erythematous vesicles and desquamation at birth, followed by progressive development of keratotic plaques with scaling across the entire body. Flexural areas exhibited friction-induced erosions. Through physical examination, the patient displayed xerosis, widespread erythematous scaly patches, and hyperkeratosis localized to the knees, elbows, dorsal hands, and feet. Additionally, scattered erosions and crusted lesions were observed on the trunk and flexural areas (Figure 1A,B). The scalp exhibited diffuse suppurative papules with patchy alopecia (Figure 1C). Immunological profiling revealed abnormalities, including elevated white blood cell count (16.01 × 10^9^/L, reference range: 4.00–12.00 × 10^9^/L) and increased absolute neutrophil count (10.60 × 10^9^/L, reference range: 1.50–7.80 × 10^9^/L), with normal eosinophil percentages.

**Figure 2:**
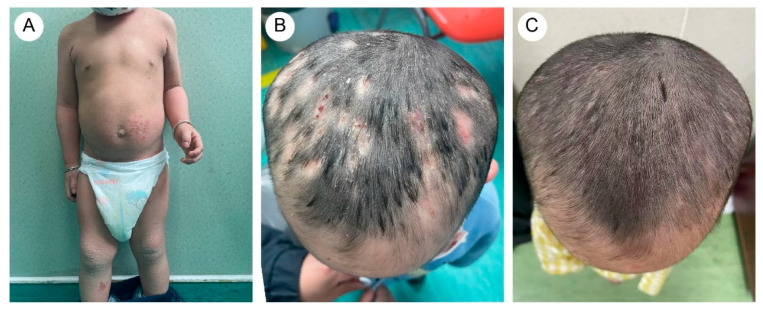
(**A**) The proband’s brother exhibited dry and scaling skin throughout the body, with excessive keratotic scales in flexural areas. There were scattered vesicles, erosion, and crusts on the trunk and lower limbs. (**B**) Mild papules and pustules were present on the scalp. (**C**) After treatment, scalp lesions improved.

The proband’s two-year-and-four-month-old half-brother from a different father demonstrated a similar but milder clinical manifestation (Figure 2A,B). Fluorescent staining of the scalp lesions from both siblings revealed sparse fungal spores and hyphae, and subsequent fungal culture confirmed T. rubrum infection. Both patients received oral terbinafine 62.5 mg once daily and topical bifonazole gel for tinea capitis. After eight weeks of therapy, scalp pustules resolved, though localized alopecia persisted (Figure 1D and Figure 2C). Follow-up immunological profiling has demonstrated normalization of all previously abnormal parameters.

### 3.2. Mutation Analysis

**Figure 3:**
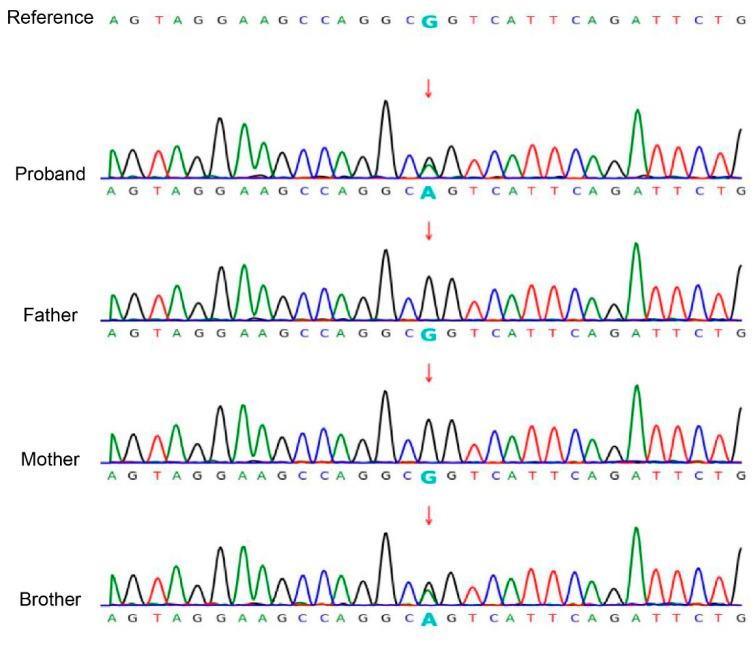
Sanger sequencing confirmed the c.466C>T: p.R156C variant in the *KRT10* (NM_000421.5) gene in the siblings. The chromatogram depicted in the figure shows the variant indicated by an arrow. Both siblings are heterozygous for the mutation, while the mother of the siblings and the father of the brother are wild type.

Based on clinical and physical findings, congenital ichthyosis was preliminarily diagnosed. Given unaffected parents, comprehensive molecular genetic analysis was conducted. The WES of the proband revealed a heterozygous *KRT10* gene mutation (NM_000421.5: c.466C>T: p.R156C). Sanger sequencing confirmed this mutation in both half-siblings, but not in either parent (wild-type) (Figure 3).

**Figure 4:**
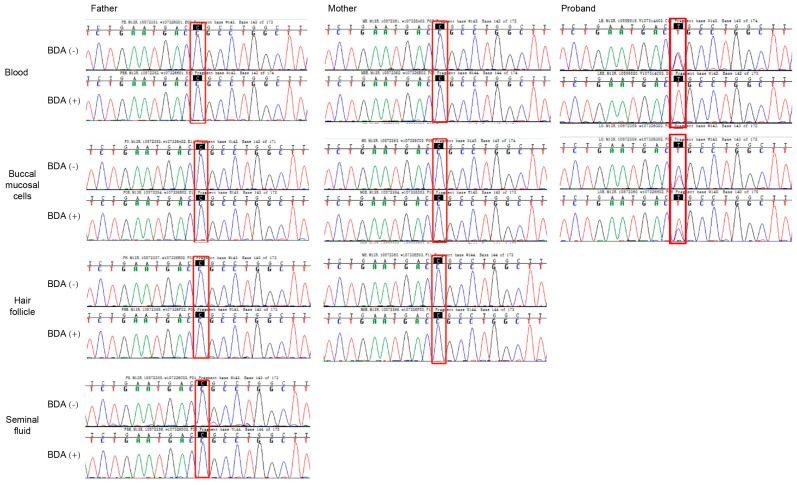
Sanger sequencing of normal and blocker displacement amplification (BDA) PCR products for mutational screening of *KRT10* in somatic cells. Results for blood, buccal mucosal cells, hair follicle, and seminal fluid from the parents of the proband. BDA (−) is normal PCR, and BDA (+) is blocker displacement amplification-based PCR.

This identical mutation in siblings with different fathers suggested maternal germline mosaicism. We further screened parental samples for mosaicism using the BDA method, which prevents false positives from pseudogene interference. BDA-based Sanger sequencing demonstrated wild-type peaks at the c.466 site in both BDA (+) and BDA (−) groups across blood, buccal mucosal cells, hair follicles, and seminal fluid samples, with no difference in signal intensity between groups (Figure 4).

In contrast, the proband’s samples exhibited the mutation in both somatic and germline cells. The BDA (−) group showed a bimodal peak (wild-type and mutant), while the BDA (+) group displayed a unimodal mutant peak (Figure 4). Specifically, at the c.466 site, the wild-type peak (blue) was effectively suppressed in the BDA (+) group, leaving only the mutant peak (red), confirming a C-to-T substitution. Similarly, in buccal mucosal cells, the mutant peak was significantly enhanced in the BDA (+) group compared to the BDA (−) group. These findings support the conclusion that the mutation in the proband was a germline variant. Based on these results, a definitive diagnosis of bullous congenital ichthyosiform erythroderma was established.

## 4. Discussion

We report a case of half-siblings with BCIE, complicated by concurrent dermatophyte infection. Scalp culture from the older sibling identified *T. rubrum*, a dermatophyte fungus. This organism secretes keratinolytic enzymes that invade the stratum corneum and keratinized tissues, leading to superficial infections such as tinea manuum, tinea pedis, and tinea capitis [17]. Children aged between 3 to 7 years old are particularly susceptible to skin fungal infections due to factors like shared toys and direct contact, facilitating fungal transmission [18]. Shared combs or caps likely contributed to the simultaneous development of tinea capitis in both siblings. Following an 8-week course of oral terbinafine, inflammation and pustules resolved in both children, though alopecia persisted. Schøsler et al. reported recurrent terbinafine resistant *T. rubrum* infection in a child with congenital ichthyosis [19]. Fortunately, our patient responded well to treatment with terbinafine, indicating they did not encounter terbinafine-resistant *T. rubrum* strains. Terbinafine is recommended for dermatophytosis due to its superior mycological, clinical, and complete cure rate compared to other antifungals [20]. Trichoscopy during initial assessment is advisable to guide management, as characteristic spiral hairs are pathognomonic for *T. rubrum* infection [21].

Ichthyosis is a disorder of keratinization, and several types of ichthyosis have been classified according to the inheritance, clinical appearance, pathological features, and systemic involvement. To date, a total of 23 ichthyosis patients have been reported with *T. rubrum* infection. Among them, the involved ichthyosis species include X-linked recessive ichthyosis, congenital ichthyosiform erythroderma, ichthyosis vulgaris, keratitis–ichthyosis–deafness syndrome, non-bullous congenital ichthyosiform erythroderma, Sjögren–Larsson syndrome, lamellar ichthyosis, and so on (Table 1). Fungal skin infections typically occur in contexts of local or systemic immunosuppression. Compromised skin barrier function, especially in inherited keratinization disorders, may increase the risk of infection. Cases of dermatophytosis (e.g., Majocchi’s granuloma in NBCIE [22], *T. rubrum*-induced hyperkeratotic lesions in ichthyosis vulgaris [23]) support this association.

To date, a total of 55 pathogenic *KRT10* variants have been reported: 35 missense, 6 nonsense, 5 frameshift, 5 deletions, and 1 synonymous mutation. We identified the recurrent missense mutation c.466C>T (p.R156C), known to disrupt the keratin filament assembly [34,35]. The elevated mutation frequency at the Arg156 residue is attributed to its location within a CpG dinucleotide motif. This sequence is prone to 5′-cytosine methylation and subsequent spontaneous deamination, leading to C>T or G>A transitions depending on the affected DNA strand [35]. Interestingly, this variant was detected in both siblings, who share the same mother but have different biological fathers. Extensive lentigo simplex, linear epidermolytic naevus, and epidermolytic naevus comedonicus were associated with c.466C>T (p.R156C) somatic mutation in *KRT10* [9]. While somatic mutations causing epidermolytic nevus in parents can involve germlines and be transmitted [36], neither parent exhibited phenotypes or detectable mutations in somatic cells or the brother’s paternal germ cells. The presence of this mutation in siblings with different fathers strongly suggests maternal germline mosaicism. However, we did not detect any mutations in the mother of the proband. Direct assessment of oocytes was not feasible. We additionally approached asymptomatic maternal relatives, as these individuals may carry comparable somatic mutations. However, genetic testing was declined due to privacy concerns. Nevertheless, we recommend comprehensive screening of family members in similar cases to detect potential mosaic mutations. Analysis of asymptomatic carriers could provide critical evidence supporting or refuting germline mosaicism.

Given the significant heredity risk implications due to parental somatic mosaicism [37], we systematically screened parental samples for low-level somatic mosaicism using the highly sensitive BDA method. While BDA, droplet digital PCR (ddPCR), and ultra-deep sequencing have all been reported for detecting parental mosaicism in genetic disorders, each has distinct technical profiles: ddPCR enables precise quantification of mutant allele frequencies and copy number variations but exhibits lower sensitivity than BDA [38], and ultra-deep sequencing offers superior sensitivity for detecting maternal mosaicism yet requires substantial sample input [39]. Due to limited sample availability, this study exclusively employed the BDA method. Regrettably, BDA-based PCR did not detect the mosaic mutations in accessible maternal tissues in our study. Our approach has limitations, including qualitative rather than quantitative verification of low-abundance mutations. Prior studies validate the sensitivity of BDA to mosaic mutations. Lin et al. reported that BDA improved testing accuracy in pseudogene-rich regions like *PKD1* [40]. Karolak et al. identified four (22%) families with *FOXF1* parental somatic mosaic single-nucleotide variants and copy number variant deletion detected in parental blood samples by BDA and ddPCR [16]. Emerging techniques like BDA-amplicon nanopore sequencing of plasma/tissue [41] and optical biosensing methods may offer enhanced sensitivity for future germline mosaicism detection [42]. We recommend integrating BDA with ddPCR, qPCR, or ultra-deep sequencing to enhance future mosaic variant detection. Collectively, our findings support the potential utility of BDA technology in genetic dermatosis diagnostics and guide reproductive counseling.

In this case, the siblings developed dermatophytosis in addition to *KRT10*-mutant BCIE, while their cohabiting parents remained unaffected. Functionally, Arg156 resides in the evolutionarily conserved 1A rod domain of KRT10, a region critical for filament assembly. Expression of basal keratin equivalents carrying Arg156Cys or Arg156His mutations in cultured keratinocytes disrupts the keratin cytoskeletal network [35]. Thus, we hypothesize that the *KRT10* gene mutation causes abnormal aggregation of keratin filaments, resulting in cytoskeletal defects and increased mechanical skin fragility, and granular layer cytolysis may reduce extracellular lipid bilayers, impairing permeability barrier function [43]. These pathological changes, blistering, hyperkeratosis, and barrier compromise, collectively increase susceptibility to fungal cutaneous infections. While the c.466C>T (p.R156C) mutation is a recognized pathogenic hotspot, conclusive functional validation is lacking. Future studies will investigate the underlying molecular mechanisms to establish a theoretical foundation for targeted therapeutic development.

## 5. Conclusions

In summary, we report a genetically confirmed case of BCIE in two non-consanguineous half-siblings. The presentation was complicated by *T. rubrum* infection with associated alopecia, which resolved following systemic antifungal therapy. This case underscores that BCIE patients may exhibit higher susceptibility to dermatophytosis, necessitating close clinical monitoring. Furthermore, this study demonstrates the utility of a BDA-based low-frequency mutation detection method, highlighting its potential to advance personalized diagnosis and management in hereditary skin diseases.

## Figures and Tables

**Table 1:** Summary of ichthyosis types complicated by *T. rubrum* infection.

Reference	Country	Age *	Type	Variant *	Dermatophyte
This study	China	5Y8M, 2Y4M	BCIE	*KRT10*	*T. rubrum*
Youssefian et al. (2022) [24]	Iran	26Y, 35Y	Ichthyosis, psoriasiform dermatitis	*PERP*	*T. rubrum,* *E. floccosum*
Agrawal et al. (2021) [25]	Indian	16Y, 19Y	X-linked recessive ichthyosis	*STS*	*T. rubrum*
Szlávicz et al. (2020) [15]	Hungary	54Y	Ichthyosis	*N.A.*	*T. rubrum*
Schøsler et al. (2018) [19]	Denmark	9Y	Congenital ichthyosiform	*N.A.*	*T. rubrum*
Ma et al. (2017) [26]	China	35Y	Keratitis–ichthyosis–deafness syndrome	*GJB2*	*T. rubrum*
Wang et al. (2015) [22]	China	11Y	Non-bullous congenital ichthyosiform erythroderma	*ALOXE3*	*T. rubrum*
Freitas et al. (2013) [13]	Brazil	87Y, 73Y, 27Y	Congenital ichthyosiform erythroderma; ichthyosis linearis circumflexa; Sjögren–Larsson Syndrome	*N.A.*	*T. rubrum*
Scheers et al. (2013) [27]	Belgium	10M	Congenital lamellar ichthyosis	*N.A.*	*T. rubrum*
Shirato et al. (2011) [28]	Dutch	35Y, 40Y	Sjögren–Larsson syndrome, followed by a return of the lamellar ichthyosis	*FALDH*	*T. rubrum*
Hoetzenecker et al. (2007) [29]	Germany	38Y	Ichthyosis vulgaris	*N.A*.	*T. rubrum*
Ludwig et al. (2001) [30]	USA	2Y	Lamellar ichthyosis	*N.A*.	*T. rubrum*
Oztürkcan et al. (1994) [31]	Tiirkiye	10Y	Non-bullous congenital ichthyosiform erythroderma	*N.A*.	*T. rubrum*
Agostini et al. (1992) [23]	Italy	31Y	Ichthyosis vulgaris	*N.A*.	*T. rubrum*
Shelley et al. (1989) [32]	USA	41Y	Congenital ichthyosiform erythroderma	*N.A*.	*Staphylococcus aureus, Pseudomonasaeruginos, T. rubrum*
Kamalam et al. (1982) [33]	Madras/India	6M, 10M	X-linked recessive ichthyosis; ichthyosis vulgaris (dominant)	*N.A*.	*T. rubrum*

* Abbreviations: Y, year; M, month; *N.A*., Not available.

## Data Availability

The raw data supporting the conclusions of this article will be made available by the authors on request.
